# Decision-Making in the Admission of Older Patients: A Thematic Analysis From Multiple-Stakeholder Perspectives

**DOI:** 10.7759/cureus.51966

**Published:** 2024-01-09

**Authors:** Ryuichi Ohta, Toshihiro Yakabe, Chiaki Sano

**Affiliations:** 1 Community Care, Unnan City Hospital, Unnan, JPN; 2 Family Medicine, Unnan City Hospital, Unnan, JPN; 3 Community Medicine Management, Shimane University Faculty of Medicine, Izumo, JPN

**Keywords:** decision-making, general medicine, qualitative research, healthcare disparities, geriatric assessment, clinical, patient admission, rural health services

## Abstract

Introduction

As rural healthcare systems grapple with an aging population, understanding the factors influencing hospital admission decisions for elderly patients is crucial. This study explores the complex interplay of medical, social, and psychological factors that shape these decisions, as perceived by multiple stakeholders, including physicians, patients, and their families.

Method

This study was conducted in Unnan City Hospital, a rural community hospital in Unnan, Japan, using a qualitative thematic analysis approach. Participants included general physicians, patients admitted more than once, and their families. One-on-one semi-structured interviews were conducted in Japanese, recorded, transcribed, and analyzed. The analysis focused on identifying themes that influence decision-making processes regarding the admission of elderly patients. The research team, comprising family medicine, public health, and community health care experts, ensured a multi-perspective approach through collaborative coding and discussion.

Results

Three primary themes emerged from the analysis: "dilemma between medical indications and social admissions," "risks and benefits of hospitalization in response to unpredictable changes in the elderly," and "social factors intertwined with the multilayered nature of hospital admission decisions." Physicians reported a conflict between their medical training and the social needs of patients, often leading to stress and negative emotions. The unpredictable health trajectories of elderly patients necessitated a nuanced risk-benefit analysis for hospitalization. In addition, social factors, such as bed availability, patient's living environment, and psychosocial contexts, significantly influenced admission decisions.

Conclusion

The study highlights the need for a more holistic approach to medical education and practice, especially in rural healthcare settings. Recognizing the complexity of factors influencing hospitalization decisions, including medical, social, and individual patient circumstances, is vital. The findings underscore the importance of integrating biopsychosocial aspects into the decision-making process for the hospitalization of elderly patients, advocating for patient-centered care that respects the unique challenges in rural healthcare environments.

## Introduction

In today's healthcare systems, the increasing number of older patients presents a significant challenge [1]. This shift toward an aging society places substantial demands on healthcare facilities and resources [2]. Healthcare providers, policymakers, and society need to understand the dynamics and implications of this trend [3].

Whether to admit older patients into healthcare facilities is complex and involves multiple facets. While established criteria for admission exist, the actual decision often requires detailed discussions among various stakeholders, including healthcare professionals, patients, their families, and relatives [4,5]. Each group contributes a unique perspective and set of concerns, making the decision-making process a delicate equilibrium [6].

Healthcare professionals, who are often at the forefront of this process, face numerous challenges [7]. They have to navigate ethical dilemmas, resource limitations, and differing opinions among stakeholders, which can lead to uncertainty and stress, affecting their ability to make timely and effective decisions [8].

Given these challenges, exploring and understanding the factors influencing admission decisions for older patients is imperative. A comprehensive examination of these factors, considering the viewpoints of all involved stakeholders, is essential [9]. Such an understanding could lead to more informed, efficient, and compassionate decision-making in healthcare settings [10,11].

This research aims to illuminate the decision-making process for admitting older patients into rural hospitals. Employing a thematic analysis approach, the study can investigate the various factors influencing these decisions, considering perspectives from medical professionals, patients, and their families [12]. This multi-dimensional approach is expected to provide an in-depth understanding of the admission process in rural healthcare settings, where resources and expertise may vary significantly from those in urban areas [13]. By identifying key themes and factors from different stakeholders' viewpoints, this study seeks to offer valuable insights for healthcare professionals, policymakers, and the broader healthcare community.

## Materials and methods

This study employed a thematic analysis grounded in relativist ontology and constructivist epistemology [14]. Hospital admission decisions vary among stakeholders, namely, physicians, patients, and their families, due to their diverse backgrounds, experiences, and perspectives. We explored these differences in the context of rural hospital admissions, focusing on stakeholder perceptions and influencing factors. This thematic analysis was framed within a qualitative research approach [15].

Setting

As of 2022, Unnan City had a population of 35,738, with 40.27% aged 65 or older. The city's primary healthcare facility, Unnan City Hospital, comprised 281 beds across acute, general, rehabilitation, and chronic care units. The hospital's internal medicine patients were collectively managed by the Department of Family Medicine [16].

Participants

The study included general physicians from Unnan City Hospital, patients admitted there more than once, and their families and relatives. The participants were selected through purposive sampling based on their backgrounds and admission reasons. Inclusion criteria encompassed willingness to participate and sufficient cognitive and physical ability to engage in interviews. Families and relatives of patients with dementia were included. Exclusion criteria involved non-consent, suspected dementia or mild cognitive impairment, and inability to participate in interviews. Informed consent was obtained from all participants. The participant group comprised seven general physicians, nine patients, seven family members, and five relatives.

Data collection

We conducted one-on-one semi-structured interviews in Japanese at Unnan City Hospital, specifically in an outpatient department. These interviews, each lasting about 60 minutes, were facilitated by the first researcher (RO), who has expertise in family medicine and public health. RO established rapport with participants before delving into a semi-structured interview guide, which included questions on previous admission experiences, decision-making criteria, challenges in decision-making, and suggestions for improving decision quality. Each interview was recorded and transcribed verbatim.

Analysis

The study utilized an in-depth inductive thematic analysis to explore themes related to social isolation, health problems, and solution strategies within rural contexts [12,15]. Interviews were meticulously recorded, transcribed verbatim, and then analyzed thoroughly. After every three interviews, RO and TY independently read the transcripts to code and identify emerging patterns and themes. This coding was iterative, deepening the understanding and refining the data with each review.

RO initially created a preliminary codebook, continually adapting based on the evolving insights from the transcripts. Concurrently, TY independently reviewed the transcripts, contributing to the coding process. The researchers then conducted extensive discussions to compare and contrast their findings, merge codes, and refine themes. This collaborative approach ensured diverse perspectives in data interpretation.

The coding and discussion process continued until saturation was reached, with no new themes emerging. At this final stage, CS, a specialist in community care, joined the analysis, adding another level of expertise and validating the themes for their applicability in community care contexts.

After finalizing the themes, the results were translated from Japanese to English. This translation was carefully undertaken to preserve the nuances and context of the original data, enabling accurate communication of the findings to an international audience.

Reflexivity

The research process was enriched by the diverse expertise within the research team, fostering collaborative interactions between researchers and participants.

The team comprised RO, a family physician and public health professional with a master’s degree in public health and family medicine; TY, a director of a non-profit organization with extensive experience in rural community support; and CS, a medical educator specializing in community health care management. Each member brought unique insights: RO provided practical experience in rural community health, TY offered a grassroots perspective from decades of supporting isolated individuals in communities, and CS contributed an academic and systematic approach to community healthcare management and education.

The team engaged in rigorous discussions to ensure an unbiased approach, constantly challenging and refining each other's ideas. This process involved exploring alternative viewpoints and reflecting on how their professional and personal experiences might influence data analysis. This reflective practice was critical in mitigating potential biases, fostering a more objective and comprehensive understanding of the study's findings.

The team's engagement with participants was marked by respect and empathy, valuing shared insights and experiences. This participatory approach facilitated a deeper understanding of the subtleties in rural healthcare perceptions and practices, enhancing the study's overall depth and relevance.

Trustworthiness

Credibility was established through prolonged engagement with participants and iterative discussions. Regular peer debriefing among the research team ensured cross-validation of findings. Detailed context descriptions, participant demographics, and methodology were provided to support transferability to other rural settings. An audit trail was maintained, reflecting the research process from data collection to analysis, thereby minimizing researcher bias. The study's dependability was reinforced by a consistent and transparent methodological approach, with a reflexive critique by the research team enhancing reliability.

Ethical consideration

The Unnan City Hospital Clinical Ethics Committee approved the study protocol (20230028).

## Results

**Figure 1:**
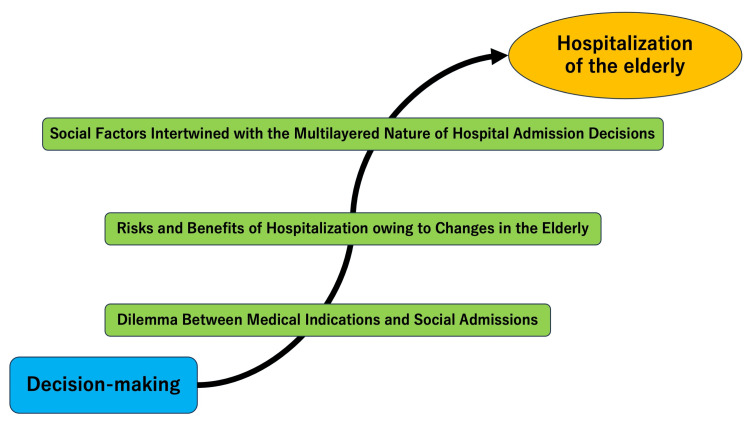
Conceptual framework of the decision-making of older patients' hospitalization

The thematic analysis revealed three key themes related to decision-making factors in the hospitalization of elderly patients in rural hospitals, as perceived by various stakeholders, including physicians, patients, and their families: "dilemma between medical indications and social admissions," "risks and benefits of hospitalization in response to unpredictable changes in the elderly," and "social factors intertwined with the multilayered nature of hospital admission decisions." Figure 1 illustrates a conceptual representation of the thematic analysis.

Dilemma between medical indications and social admissions 

Physicians are continually educated on the medical criteria for hospitalization. However, the participants frequently encountered cases where these criteria were inadequate, prompting them to reconsider the notion of "medical indications" for admission. A 28-year-old physician expressed, “During my residency, I learned about hospitalization indications, but I found them less effective in addressing the complex issues faced by elderly patients in rural areas, who often have challenges beyond medical concerns.” This discrepancy highlights the relativity of hospital admission criteria. Oftentimes, patients with severe symptoms that justify hospitalization choose not to be admitted due to financial or familial considerations, altering the traditional indicators for admission. Another physician, 31 years old, stated, “Balancing medical and social elements complicates the decision to admit older patients. As physicians, we cannot base our decisions solely on the patient's symptoms.”

In cases where physicians perceive no medical justification for a patient's admission, they might experience negative emotions toward the patient or family’s desire for hospitalization. Some physicians exhibit negative attitudes in these situations, leading to strained relations with patients and their families. A 76-year-old patient recalled, “I remember the reluctance in the physicians’ faces when I sought admission. They might have had their criteria for hospitalization, but they seemed unaware of my struggles with fatigue and appetite. It made me feel misunderstood.” 

Conversely, physicians’ training in medical hospitalization can lead to a dismissal of the diverse, real-world factors influencing admission decisions. Even when acknowledging these factors, some physicians' inherent biases make them hesitant to approve admissions based on social needs. A 28-year-old physician admitted, “I recognize that there are various reasons for hospitalization in practice. However, I often feel negatively about admitting patients for social reasons, perhaps due to my medical training.”

Risks and benefits of hospitalization in response to unpredictable changes in the elderly

The progression of symptoms in elderly patients can be unpredictable, significantly impacting hospitalization decisions. Physicians have encountered numerous instances where patients' conditions deteriorated unexpectedly after initially appearing stable, leading them to question the absolute criteria for hospital admission at the time of the clinic visit. A 27-year-old physician shared, “Predicting the clinical course of elderly patients is challenging due to their vulnerability. I’ve made several errors where I misjudged their condition, resulting in critical outcomes.” Especially in elderly patients, the likelihood of developing complications like delirium or frailty due to hospitalization is a concern. This necessitates a careful evaluation of the risks and benefits of admitting them. A 36-year-old physician mentioned, “Rash decisions regarding admission can lead to various complications in older patients. We have to seriously consider the risks involved in admitting them.”

When no one can provide home care, hospitalization often becomes inevitable. The participants reflected on their experiences and believed that the necessity of hospitalization and the potential for complications significantly influenced their admission decisions. A 77-year-old patient said, “I used to care for myself, but as I aged, my confidence in managing my health and recognizing worsening symptoms diminished, prompting me to seek hospitalization.” Despite understanding the risks associated with hospitalization, such as nosocomial infections and delirium, some families opt for hospitalization due to the fear of acute changes in the condition of elderly patients and the exacerbation of diseases, which could prolong the duration of the hospital stay. A 45-year-old family member expressed, “I worried about my father’s condition deteriorating due to admission. However, the concern about his condition worsening at home outweighed the risks of hospitalization.”

Social factors intertwined with the multilayered nature of hospital admission decisions

The bed occupancy rate in rural hospitals fluctuates seasonally and annually, influenced by the prevalence of infections and cardiovascular diseases. At times, ineffective bed management means those in need cannot be admitted. A 30-year-old physician explained, “During winter, the influx of patients with influenza and pneumonia is overwhelming. I prefer to hospitalize some patients, but due to bed management challenges, I have to treat them as outpatients, mindful of the risk of their condition worsening.” On the other hand, when bed vacancies are high, there can be an implicit push to increase admissions, with physicians' thresholds for hospital admission being swayed by the environment of the medical institution. This sometimes results in unconscious pressure to fill hospital beds. A 27-year-old physician reflected, “Bed occupancy rates are not a critical issue in my public hospital. However, I often feel that vacant beds should be utilized, driven by a sense that leaving them empty is wasteful.”

The patient's living environment and the potential for rapid symptom progression are considered when assessing the need for hospitalization. Rapidly worsening symptoms can pose a significant risk to vulnerable patients living far from medical facilities. An 89-year-old patient shared, “Living remotely, I feared rapid progression and deterioration of my symptoms. Although it may not have been strictly necessary, I sought hospitalization when I suffered from pneumonia.” The dynamics between physicians, patients, and families can influence the threshold for hospitalization. Negative sentiments can impede necessary admissions. A 61-year-old family member of a patient recounted, “My mother had a severe infection, but the physician initially refused admission. Given the harsh winter conditions that make travel difficult, social factors should be considered in the hospitalization of older patients.” The rise in the number of solitary elderly individuals heightens anxiety among patients and their families about the worsening of medical conditions. Physicians find themselves wrestling with the decision to hospitalize, balancing medical necessity against these concerns.

## Discussion

This study has elucidated three principal themes related to the decision-making process for hospital admissions in rural hospitals, as perceived by various stakeholders, including physicians, patients, and their families. These themes encapsulate the intricate interplay between medical indications, social factors, and the unpredictability inherent in treating elderly patients.

First, physicians encounter a profound dilemma between medical indications and the need for social admissions, which should be mitigated through revised medical education [17]. Their medical training, predominantly focused on clinical criteria, often needs to be adjusted to match the complex social realities of patient care in rural contexts [18]. This conflict leads to a dichotomy where physicians struggle to reconcile their professional knowledge with the nuanced realities of their patient's lives, particularly those of the elderly [19]. This study revealed instances where physicians felt challenged by cases that did not fit the textbook criteria for hospitalization but were compelled by the patient's social circumstances. To mitigate their potential conflicts, medical education should include the reality of older patients’ hospitalization affected by various factors [20].

Second, the risk-benefit analysis associated with hospitalizing elderly patients emerged as a crucial consideration. Older patients are particularly vulnerable to complications during hospital stays, yet certain medical conditions necessitate admission [21,22]. This complexity is heightened by the unpredictable nature of elderly patients' health, which often changes rapidly and unexpectedly. Not only aging but multimorbidity and polypharmacy can modify their physiological conditions, so older patients’ clinical courses can differ from younger patients [23]. The participants in this study reflected on cases where the clinical course of an elderly patient was difficult to predict, leading to critical situations due to delayed or premature hospitalization. Such experiences may make the decision-making of hospitalization of older patients challenging and the threshold of hospitalization lower in rural hospitals.

Finally, social factors significantly influence hospital admission decisions. These factors are intricately linked with the multilayered nature of decision-making in hospital admissions [24]. This study found that bed occupancy rates, the incidence of diseases, and patients' personal experiences and environments all play a role in determining hospitalization needs. Physicians with prior knowledge of medical admission could not help to follow the social demands of the organizations by which they are hired [25]. Patients and families tend to feel frustrated but accept the situation as if they can do nothing [26]. The threshold for hospitalization is influenced not just by medical criteria but also by the broader context of the patient's living conditions, their proximity to medical facilities, and their personal and family circumstances.

In light of these findings, medical education must be adapted to address the balance between medical and social conditions in patient care regarding hospitalization. Various social factors affect patients’ medical conditions, called social determinants of health (SDH) [27]. As this study shows, patients' living conditions and relationship with their physicians, relatives, and families can affect their health conditions regarding admissions as SDH. Especially in rural contexts, older patients can be independent, but social interaction can decrease because of aging [28]. Medical professionals should be trained to recognize the importance of social factors and SDH in hospitalization decisions and to mitigate the negative feelings and stress that may arise from these complex situations [29]. Education should emphasize that hospitalization decisions, particularly for elderly patients in rural areas, often require a departure from strict medical criteria, requiring a more holistic approach that considers the patient's overall well-being [30].

The study, however, has limitations. Its scope, confined to a single rural community hospital, may limit the generalizability of its findings. The research involved a detailed interactive process and decision-making analysis based on iterative data collection and in-depth context descriptions to address this. Data were collected from multiple stakeholders to understand the factors influencing hospital admissions in rural settings. To enhance the study's reliability, iterative data analysis was conducted over an extended period, and two authors coded the data with different perspectives and backgrounds. A third researcher reviewed the coding, concepts, and themes for triangulation, further validating the study's findings.

## Conclusions

The decision-making process for the hospitalization of elderly patients in rural settings is a complex interplay of medical, social, and personal factors. Physicians, influenced by their medical training, often face a dilemma when the standard medical indications for hospitalization conflict with the social realities of patient care. Stakeholders are required to navigate the risks and benefits of hospitalizing elderly patients, who are prone to complications and unpredictable health changes. Social factors, including the hospital environment and the psychosocial contexts of patients and families, also significantly influence these decisions. To effectively address these challenges, a collaborative and realistic approach is needed, one that respects the multifaceted nature of healthcare in aging societies. Medical education should evolve to embrace a holistic view of patient care, recognizing that there are no absolute standards for correct hospitalization, especially in the context of elderly patient care in rural hospitals.

## References

[1] Qi X, Li Y, Hu J (2023). Prevalence of social frailty and its associated factors in the older Chinese population: a national cross-sectional study. BMC Geriatr.

[2] Cudjoe TK, Prichett L, Szanton SL, Roberts Lavigne LC, Thorpe RJ Jr (2022). Social isolation, homebound status, and race among older adults: findings from the National Health and Aging Trends Study (2011-2019). J Am Geriatr Soc.

[3] Tang N, Eisenberg J, Meyer G (2004). The roles of government in improving health care quality and safety. Jt Comm J Qual Saf.

[4] Sommers LS, Marton KI, Barbaccia JC, Randolph J (2000). Physician, nurse, and social worker collaboration in primary care for chronically ill seniors. Arch Intern Med.

[5] Ohta R, Sano C (2023). The effectiveness of family medicine-driven interprofessional collaboration on the readmission rate of older patients. Healthcare (Basel).

[6] Lancaster G, Kolakowsky-Hayner S, Kovacich J, Greer-Williams N (2015). Interdisciplinary communication and collaboration among physicians, nurses, and unlicensed assistive personnel. J Nurs Scholarsh.

[7] Eika M, Dale B, Espnes GA, Hvalvik S (2015). Nursing staff interactions during the older residents' transition into long-term care facility in a nursing home in rural Norway: an ethnographic study. BMC Health Serv Res.

[8] Einav S, Benoit DD (2019). Focus on ethics of admission and discharge policies and conflicts of interest. Intensive Care Med.

[9] Henke RM, Karaca Z, Jackson P, Marder WD, Wong HS (2017). Discharge planning and hospital readmissions. Med Care Res Rev.

[10] Chan EY, Samsudin SA, Lim YJ (2020). Older patients' perception of engagement in functional self-care during hospitalization: A qualitative study. Geriatr Nurs.

[11] Geyskens L, Jeuris A, Deschodt M, Van Grootven B, Gielen E, Flamaing J (2022). Patient-related risk factors for in-hospital functional decline in older adults: a systematic review and meta-analysis. Age Ageing.

[12] Braun V, Clarke V (2006). Using thematic analysis in psychology. Qual Res Psychol.

[13] Williams S, Fernandes G (2023). Cutting edge research? Realistic expectations of priorities, scope and engagement comment on "'we're not providing the best care if we are not on the cutting edge of research': a research impact evaluation at a regional Australian hospital and health service". Int J Health Policy Manag.

[14] Berryman DR (2019). Ontology, epistemology, methodology, and methods: information for librarian researchers. Med Ref Serv Q.

[15] Vaismoradi M, Turunen H, Bondas T (2013). Content analysis and thematic analysis: Implications for conducting a qualitative descriptive study. Nurs Health Sci.

[16] Ohta R, Ryu Y, Sano C (2021). Family medicine education at a rural hospital in Japan: impact on institution and trainees. Int J Environ Res Public Health.

[17] Farmer J, Bourke L, Taylor J, Marley JV, Reid J, Bracksley S, Johnson N (2012). Culture and rural health. Aust J Rural Health.

[18] Somporn P, Walters L, Ash J (2018). Expectations of rural community-based medical education: a case study from Thailand. Rural Remote Health.

[19] Ohta R, Ryu Y, Katsube T, Moriwaki Y, Otani J (2019). Students' perceptions of general medicine following community-based medical education in rural Japan. J Gen Fam Med.

[20] Moll-Jongerius A, Langeveld K, Helmich E, Masud T, Kramer AW, Achterberg WP (2023). Becoming a physician for older patients: exploring the professional identity formation of medical students during a nursing home clerkship. A qualitative study. BMC Med Educ.

[21] Chen X, Mao G, Leng SX (2014). Frailty syndrome: an overview. Clin Interv Aging.

[22] Ohta R, Weiss E, Mekky M, Sano C (2022). Relationship between dysphagia and home discharge among older patients receiving hospital rehabilitation in rural japan: a retrospective cohort study. Int J Environ Res Public Health.

[23] Stafford G, Villén N, Roso-Llorach A, Troncoso-Mariño A, Monteagudo M, Violán C (2021). Combined multimorbidity and polypharmacy patterns in the elderly: a cross-sectional study in primary health care. Int J Environ Res Public Health.

[24] Covinsky KE (2013). Hospitalization in older persons: not just a medical outcome, a social outcome as well: comment on "Elder abuse as a risk factor for hospitalization in older persons". JAMA Intern Med.

[25] Kunzler AM, Helmreich I, Chmitorz A, König J, Binder H, Wessa M, Lieb K (2020). Psychological interventions to foster resilience in healthcare professionals. Cochrane Database Syst Rev.

[26] Kandasamy D, Platts-Mills TF, Shah MN, Van Orden KA, Betz ME (2018). Social disconnection among older adults receiving care in the emergency department. West J Emerg Med.

[27] Spruce L (2019). Back to basics: social determinants of health. AORN J.

[28] Naito Y, Ohta R, Sano C (2021). Solving social problems in aging rural Japanese communities: the development and sustainability of the Osekkai Conference as a social prescribing during the COVID-19 pandemic. Int J Environ Res Public Health.

[29] Ohta R, Naito Y, Sano C (2023). Mutually supportive and inclusive societies driven by community social workers in japan: a thematic analysis of Japanese comics. Geriatrics (Basel).

[30] Doobay-Persaud A, Adler MD, Bartell TR (2019). Teaching the social determinants of health in undergraduate medical education: a scoping review. J Gen Intern Med.

